# Rapid Detection of Microparticles Using a Microfluidic Resistive Pulse Sensor Based on Bipolar Pulse-Width Multiplexing

**DOI:** 10.3390/bios13070721

**Published:** 2023-07-09

**Authors:** Ruiting Xu, Leixin Ouyang, Rubia Shaik, Heyi Chen, Ge Zhang, Jiang Zhe

**Affiliations:** 1 Department of Mechanical Engineering, University of Akron, Akron, OH 44325, USA; rx7@uakron.edu (R.X.); lo10@uakron.edu (L.O.); hc77@uakron.edu (H.C.); 2 Department of Biomedical Engineering, University of Akron, Akron, OH 44325, USA; rs169@uakron.edu (R.S.); ge10@uakron.edu (G.Z.)

**Keywords:** resistive pulse sensor, signal multiplexing, bipolar pulse, high throughput, particle counting, microfluidics, iterative cancellation

## Abstract

Rapid and accurate analysis of micro/nano bio-objects (e.g., cells, biomolecules) is crucial in clinical diagnostics and drug discovery. While a traditional resistive pulse sensor can provide multiple kinds of information (size, count, surface charge, etc.) about analytes, it has low throughput. We present a unique bipolar pulse-width, multiplexing-based resistive pulse sensor for high-throughput analysis of microparticles. Signal multiplexing is enabled by exposing the central electrode at different locations inside the parallel sensing channels. Together with two common electrodes, the central electrode encodes the electrical signal from each sensing channel, generating specific bipolar template waveforms with different pulse widths. Only one DC source is needed as input, and only one combined electrical output is collected. The combined signal can be demodulated using correlation analysis and a unique iterative cancellation scheme. The accuracy of particle counting and sizing was validated using mixtures of various sized microparticles. Results showed errors of 2.6% and 6.1% in sizing and counting, respectively. We further demonstrated its accuracy for cell analysis using HeLa cells.

## 1. Introduction

Bioanalysis can benefit significantly from the utilization of various types of micro/nano particles, which possess unique properties such as a large surface-to-volume ratio and excellent biocompatibility [1,2]. Detection of these particles in a solution can provide valuable information that reflects the biotarget situations across a broad spectrum of applications, including biomedical research, public health, and food safety [3,4,5]. Therefore, development of portable, cost-effective, and efficient devices for particle detection is crucial for bioanalysis.

Resistive pulse sensing (RPS) is an established technology used to rapidly detect nano/micro-scaled particles [6,7,8]. A typical RPS system comprises two electrodes placed on each side of a sensing channel, filled with a conducting electrolyte solution. The passage of a single particle causes a temporary change in the electrical resistance of the sensing channel, which generates a current/voltage pulse picked up by the pair of electrodes [9]. The magnitude and duration of the pulse are dependent on the particle size and shape, allowing for the determination of both sizes and concentrations of particles [10,11]. This technique is commonly used in biomedical and nanotechnology research for applications including cell counting [12,13], nanoparticle characterization [14,15], and biomolecular detection [16,17]. It is a versatile and non-destructive method that enables real-time monitoring of individual particles as they traverse the sensing channel, making it a valuable tool for particle analysis with a single-particle resolution. To increase the throughput of detection, RPS devices with multiple channels using space division multiplexing [18], CODES multiplexing [19,20], geometry multiplexing [21], and frequency division multiplexing [22] have been developed. The space multiplexing method [18] has demonstrated high-throughput parallel analysis with eight sensing channels, each equipped with individual measurement electronics. However, as the number of channels increases, it becomes impractical to implement individual detection electronics for each channel. CODES division [20] and geometry multiplexing [21] method have been proposed, but they require complex, unique patterns of microelectrodes or microchannel geometry for each sensing channel to generate specific waveform patterns; both methods are difficult to apply in nanoscale particle detection. Additionally, the frequency-division multiplexing method [22] requires the device be operated in the resistance-dominant frequency region; only a limited number of sensing channels can be used, resulting in limited scalability.

Here, we introduce a bipolar pulse-width multiplexing microfluidic sensor that provides a simple and scalable solution for high-throughput micro/nano particle counting. The sensor does not need complex electrodes or geometry; specific waveforms can be generated by three electrodes operated in tandem using only one DC power source. Microparticle presence and sizes can be determined through correlation analysis and an iterative cancellation scheme, even when multiple particles are present in the sensing channels.

## 2. Materials and Methods

### 2.1. Materials

Polystyrene microparticles (15 μm, product #74964 and 20 μm, product #74491, Sigma-Aldrich, St. Louis, MO, USA) were used for demonstrating the multiplexed sensor, while 3-aminopropyl)triethoxysilane (APTES, 99%, product #440140, from Sigma-Aldrich) was used to enhance the bonding between photoresist (SU-8 6002) and polydimethylsiloxane (PDMS). Dulbecco’s phosphate-buffered saline (DPBS, 1×, product# MT21031CV, Thermo Fisher Scientific) was used to prepare the particle solution. Silver foil (0.5 mm thick, 99.9% metals basis, 25 × 25 mm, Catalog #AA39181FF) was purchased from Fisher Scientific, serving as the central electrode. 

Human negroid cervix epithelioid carcinoma cells (HeLa, product# 93021013), minimum essential medium eagle, with ear (EMEM, product# M2279), L-glutamine solution Bioxtra (product# G7513), MEM non-essential amino acid (NEAA, product# M7145), fetal bovine serum (FBS, product# F0926), and 0.25% trypsin-EDTA solution (product# T4049) were purchased from Sigma-Aldrich. Dulbecco’s phosphate-buffered salt solution 1× (DPBS, Cat. No: MT21031CV), penicillin–streptomycin (Cat. No: 15-140-122), trypan blue solution 0.4% (Cat. No: BW17-942E), and saline solution (Cat. No. L97815) were obtained from Fisher Scientific. Briefly, HeLa cells were cultured in complete EMEM medium containing 2 mM L-glutamine solution, 1% NEAA, 10% FBS, and 1% penicillin. The cells were seeded at a density of 3500 cells/cm^2^ in a T75 flask and incubated at 37 °C. The following day, the media was changed and then changed every other day until the flask reached about 90% confluency. To harvest the cells, they were washed once with 1× DPBS, and then 4 mL of 0.25% trypsin–EDTA solution was added and incubated at 37 °C for 5 min. Additional 8 mL of media was added, and the cells were pelleted in a 4 °C centrifuge at 220 g for 5 min. The cells were counted after staining with 0.4% trypan blue solution and then resuspended in 1× DPBS to a final working concentration of 10^5^ cells/mL followed by high-throughput counting.

### 2.2. Sensing Principle

**Figure 1:**
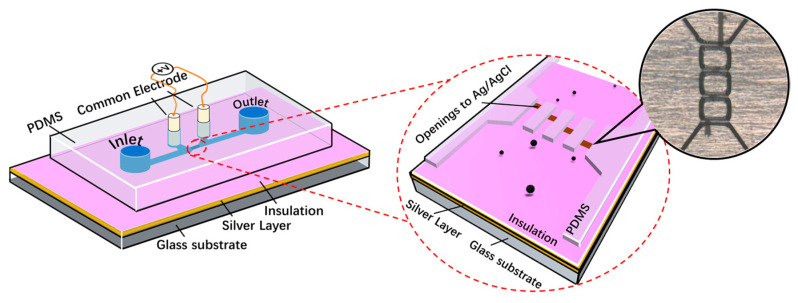
Left: illustration of the bipolar pulse-width multiplexing RPS device with four parallel sensing channels. Right: an enlarged image of the four parallel sensing channels.

A multichannel RPS device with four parallel sensing channels was designed to demonstrate the bipolar pulse-width multiplexing method for high-throughput microparticle counting, as shown in Figure 1. The microfluidic device comprised (1) four microfluidic channels made of PDMS; (2) one Ag/AgCl central electrode embedded on the bottom of each microchannel separating each sensing channel to two sections at different locations; (3) two Ag/AgCl common electrodes positioned on each side of the sensing channels; and (4) a pair of inlet and outlet reservoirs. The central electrode was formed through the following steps: (a) bonding a 0.5 mm silver layer to the glass substrate; (b) coating an insulating SU-8 layer on top of the Ag layer; (c) patterning four openings at different positions on the bottom of the four sensing channels, to expose the Ag electrode to the electrolyte; (d) treating the Ag layer with an electrochemical reaction to convert the exposed Ag to an Ag/AgCl electrode (see details in Section 2.3).

**Figure 2:**
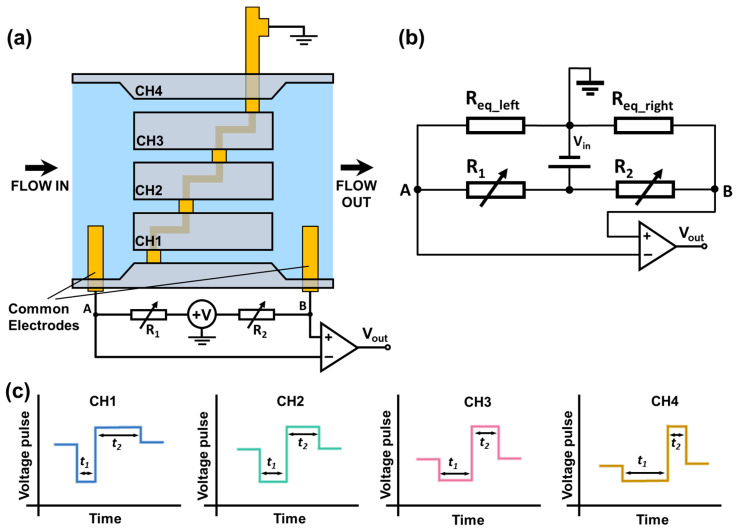
Schematic of the bipolar pulse-width multiplexing RPS device. (**a**) Illustration of the measurement set-up. (**b**) Diagram of a circuit that measures the resistive pulses generated by particles transiting through the sensing channels. (**c**) Illustration of bipolar voltage pulses/waveforms when one particle transits through different sensing channels (Blue: channel 1, Green: channel 2, Pink: channel 3, Yellow: channel 4). The bipolar pulses generated in different channels have different pulse widths t_1_ and t_2_.

A bridge circuit is used to monitor the resistance changes of sensing channels, as shown in Figure 2a,b. R_eq_left_ and R_eq_right_ represent the total resistance of the left sensing section and the right sensing section separated by the central electrode. R_1_ and R_2_ are the external adjustable resistors that form a Wheatstone bridge with R_eq_left_ and R_eq_right_. A 1 V DC input voltage (V_in_) is applied to the circuit. When a particle passes through the sensing channels (left section and right section), it induces a resistance change in R_eq_left_ or R_eq_right_, causing a differential voltage between A and B. The voltage change is detected as the electrical output signal (V_out_). When the particle passes through the left sensing section, R_eq_left_ increases while R_eq_right_ remains the same, causing a drop in voltage output. Similarly, when the particle passes the right sensing section, R_eq_right_ increases, causing a rise in voltage output. Hence, when a particle passes through each sensing channel consisting of two consecutive sensing sections, it generates a bipolar voltage pulse. Because the central electrode separates each sensing channel at different positions, the resistance/voltage drop and rise in each sensing channel occur differently when a particle passes through each sensing channel. Hence, the bipolar output from each sensing channel has a unique waveform (with different pulse widths t_1_, t_2_), as illustrated in Figure 2c. If multiple particles are present in different sensing channels, the overall voltage output measured across the common electrodes would be a combination of individual signals from each sensing channel. From the correlation analysis with the standard waveforms of each channel (generated by a fixed-size particle) and iterative cancellation scheme, we can identify in which channels the particles are transiting, as well as the count and size of particles passing each sensing channel. The magnitudes of the standard waveforms can be used to determine the sizes of the particles.

### 2.3. Device Fabrication

To fabricate the microfluidic device, the standard soft lithography method was utilized. First, the SU-8 2025 (MicroChem, Newton, MA, USA) mold was created, which included the four parallel sensing channels, two detecting reservoirs for the common electrodes, and one inlet/outlet reservoir. A PDMS (polydimethylsiloxane, Sylgard 184, Dow Corning) slab was then made by pouring the PDMS onto the top of the SU-8 mold, followed by degassing and curing the PDMS at 70 °C for 2 h. To fabricate the substrate with embedded Ag/AgCl, the 0.5 mm silver foil was bonded to a glass slide and was subsequently coated with SU-8 6002 (MicroChem, Newton, MA, USA). The insulating SU-8 layer was then subjected to a soft bake at 95 °C for 3 min and exposed to UV-light to pattern four openings for the Ag layer. After a post-bake (95 °C for 2 min) and development by SU-8 developer, four openings were created on the insulation layer. The insulated silver foil with four openings was immersed in AgCl solution, and DC power (5 V) was applied between the silver foil (positive electrode) and the silver rod (negative electrode) to facilitate the conversion of Ag to AgCl at the four openings. Next, the PDMS slab was punched to create inlet/outlet reservoirs and electrode holes for common electrodes. The PDMS slab was treated with air plasma (200 mTorr, 50 W, 50 s). The treated PDMS slab and SU-8 coated silver layer were immersed in 5% v/v aqueous APTES solution for 20 min, washed with DI water, and dried. Under a microscope, the two parts (PDMS slab and the substrate with four openings) were aligned and placed on a hotplate at 90 °C for over 30 min to form an irreversible bond. The nominal dimensions of the sensing channels were 120 μm (length), 40 μm (width), and 35 μm (height). The dimensions of the sensing channels were measured by a surface profilometer (Dektak 150, Veeco Instrument, Plainview, NY, USA): 122.4 ± 0.5 μm (length), 40.8 ± 0.3 μm (width), and 35.7 ± 0.2 μm (height).

## 3. Results

**Figure 3:**
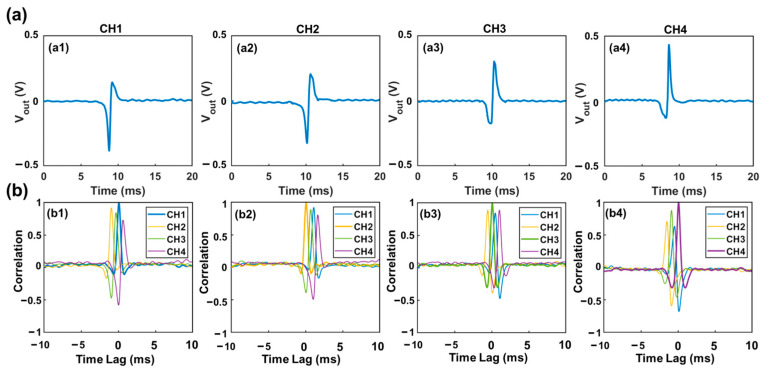
Illustration of correlation analysis (**a**) Voltage template waveforms generated by a 15 µm particle transiting through each sensing channel. (**b**) Correlation analysis between the combined voltage output and the four different template waveforms. The maximum correlation coefficient can be used to identify the presence of a particle in a specific sensing channel. The starting time of the signal can be determined based on the time lag it exhibits.

15 µm polystyrene particles suspended in DI water were used to generate the specific voltage template waveforms from each sensing channel. Typical waveforms generated in all sensing channels were recorded separately, as shown in Figure 3a. These waveforms were used as template waveforms to correlate with the combined output signal of the device. When a particle passes one specific sensing channel (e.g., channel 1), the combined signal must contain a component that is highly similar to the waveform of this channel (e.g., Channel 1). Hence a large correlation coefficient (maximum value > 0.4) would be generated. In other words, a high correlation coefficient indicates a high similarity between the combined signal and the template waveform due to the passage of a particle through this sensing channel. Visa versa, a lower correlation coefficient (maximum value < 0.4) indicates low similarity between the two signals, or no particle passage through this specific sensing channel. Thus, by correlating the combined output signal with the four standard template waveforms, the presence of particles in each channel can be determined based on the maximum correlation coefficient (Figure 3b). The template waveform shows a higher correlation coefficient with itself than other template waveforms. Also, the starting and ending time of each pulse (representing the entry and exit of each particle) can be determined using the time lag [23]. Prior studies indicated that the correlation coefficient of less than 0.4 suggests a weak similarity between two signals [24,25]. Thus, here we set correlation coefficient of 0.4 as a threshold to judge whether the detected signal contains a specific waveform. In addition, a smaller particle passing through a sensing channel would generate a highly similar waveform but with a reduced magnitude.

**Figure 4:**
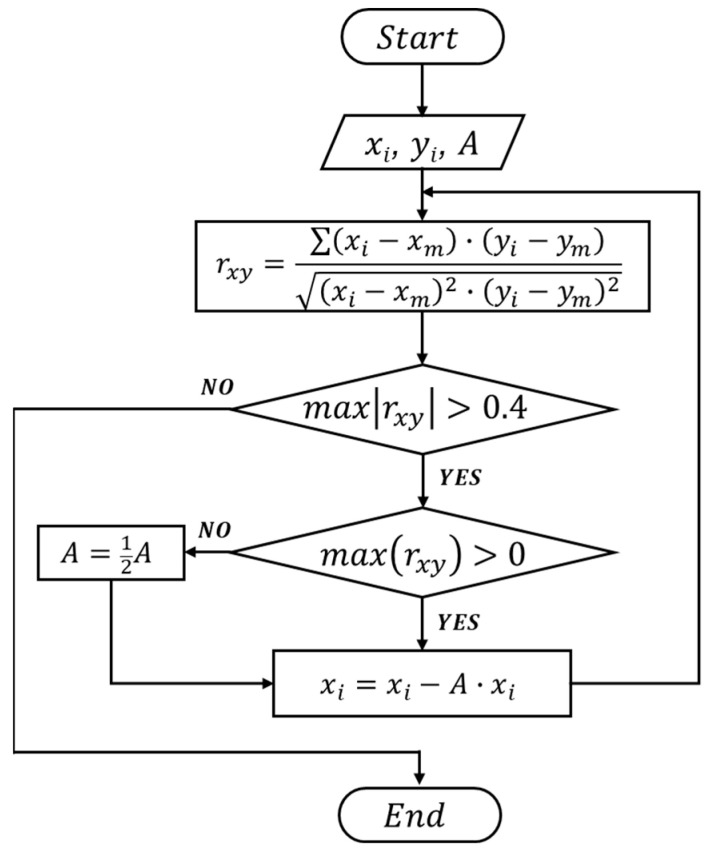
Flowchart of the iterative cancellation procedure to demodulate the detected signal (combined signal from all sensing channels).

The correlation coefficient can be calculated by $r_{xy}=\frac{\sum \left(x_{i}-x_{m}\right)\cdot \left(y_{i}-y_{m}\right)}{\sqrt{{\left(x_{i}-x_{m}\right)}^{2}\cdot {\left(y_{i}-y_{m}\right)}^{2}}}$, where $x_{i}$, $y_{i}$ represents each set of data (i.e., the combined output signal and a specific template waveform), and $x_{m}$, $y_{m}$ is the average of respective data value [25,26]. A maximum correlation coefficient > 0.4 between two sets of data indicates the combined signal contains a specific waveform (or the presence of a particle in a specific channel where the waveform is generated). To extract the desired waveform from the combined signal, an iterative cancellation scheme is employed, as illustrated in Figure 4. The sequence is as follows: After the correlation analysis, if the maximum correlation coefficient is positive and larger than 0.4 (indicating the combined signal contains the corresponding waveform), a fraction (represented by ‘A’, e.g., 0.8×) of the template waveform is subtracted. The remaining signal is then correlated with all template waveforms in the next round. During the correlation-cancellation procedure, if the maximum correlation coefficient is larger than 0.4 but negative, it indicates the template signal was over-subtracted. Hence a smaller fraction of subtraction (e.g., subtraction of $\frac{1}{2}A$× waveform) should be used. The procedure is repeated until all correlation coefficients with all template waveforms are less than 0.4. The total magnitude of all subtractions can be used to calculate the size of particles, as the particle volume is proportional to the magnitude of the detected signal [9,27].

### 3.1. Validation of Bipolar Pulse-Width Multiplexing Method

**Figure 5:**
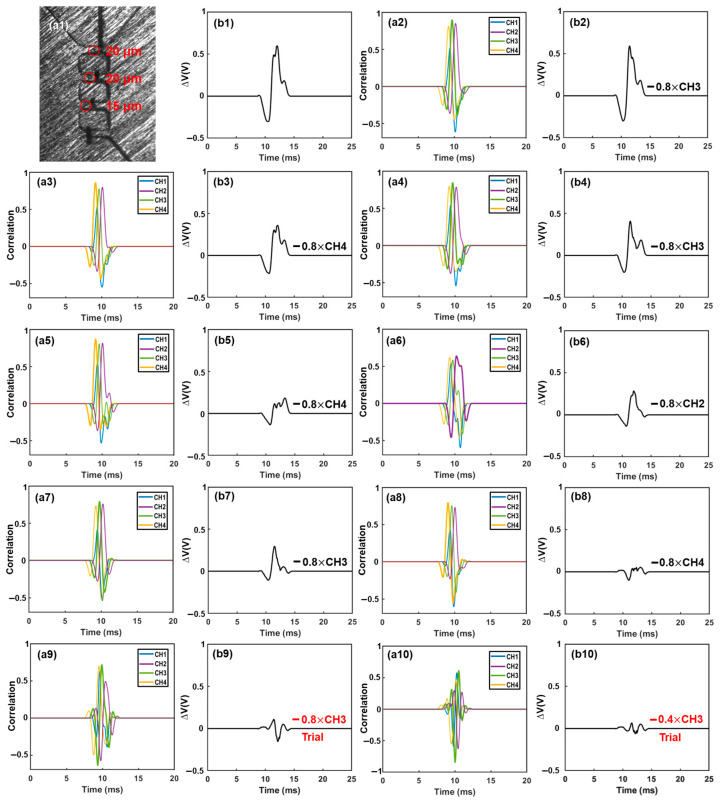

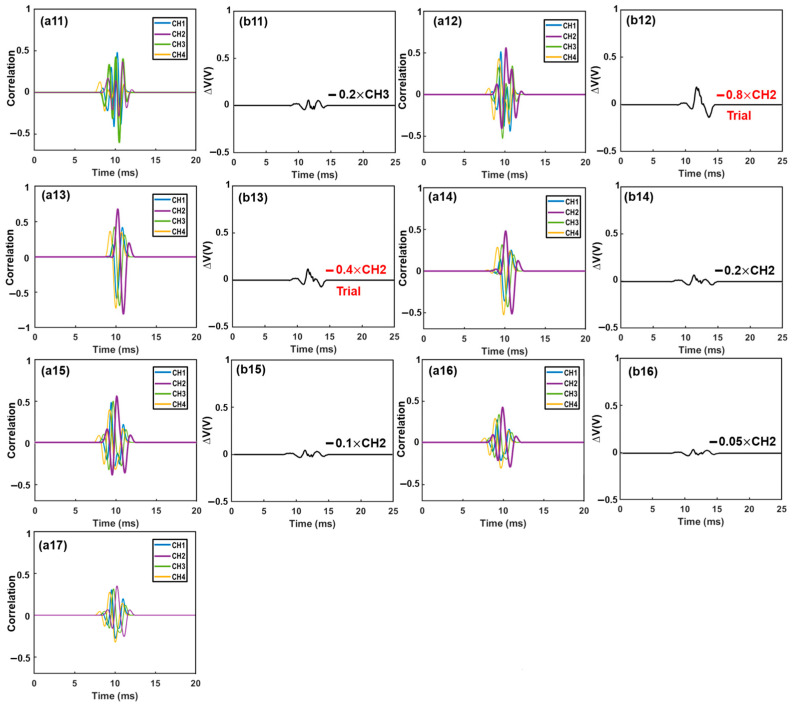
Iterative cancellation procedures for demodulation of the combined electrical signal. The combined signal was generated by three differently sized microparticles. The desired waveform was determined based on the correlation coefficient and then extracted from the remaining signal with a specific amplitude (0.8×, 0.4×, 0.2×, 0.1×, etc.). Initially, subtraction of a waveform with an amplitude of 0.8× was performed. If the correlation coefficient reached a highly negative value (indicating an over-subtraction occurred), subsequent subtractions of smaller amplitudes (e.g., 0.4×, 0.2×, 0.1×, 0.05×) were used to replace the 0.8× subtraction. Subtractions marked in red were trial subtractions that resulted in over-subtraction (highly negative correlation coefficient) and were not executed. The actual subtractions, marked in black, were performed on the combined signal ((**a1**)–(**a17**): correlation analysis between the combined signal or remaining signal and the four template waveforms. (**b1**): combined signal. (**b2**)–(**b16**): remaining signals after each subtraction).

To demonstrate the principle of the bipolar pulse-width multiplexing method, we analyzed a case where multiple particles were present in different sensing channels, as shown in Figure 5. The particle solution consisted of two different sized polystyrene microparticles (15 ± 0.2 μm and 20 ± 0.3 μm). For validation purpose, a high-speed camera (MU043M-FL, United Scope LLC, Irvine, CA, USA) was employed to record the particle transits through the sensing channel. Template waveforms were generated by a 15 μm microparticle passing through each individual sensing channel, as depicted in Figure 3a. Correlation analysis was first performed between the combined signal and the template waveforms to identify the sensing channel through which the particle had passed.

Figure 5 illustrates the demodulation procedures when multiple particles were present in different sensing channels (Figure 5(a1)). The recorded electrical signal is shown in Figure 5(b1). First, the recorded signal was correlated with all template waveforms. The maximum correlation coefficient (*max*(*r_xy_*) > 0.4) occurred in channel 3 (Figure 5(a2)), indicating the presence of one particle in this channel. Subsequently, a 0.8× waveform of channel 3 was subtracted from the combined signal, as depicted in Figure 5(b2). The correlation analysis was then repeated with all template waveforms, and the maximum correlation coefficient (*max*(*r_xy_*) > 0.4) was found in channel 4 (Figure 5(a3)). Consequently, a 0.8× waveform of channel 4 was subtracted from the remaining signal (Figure 5(b3)). This interactive cancellation procedure was continued. An 0.8× subtraction of waveform 3 (Figure 5(b4)), an 0.8× subtraction of waveform 4 (Figure 5(b5)), an 0.8× subtraction of waveform 2 (Figure 5(b6)), an 0.8× subtraction of waveform 3 (Figure 5(b7)), and an 0.8× subtraction of waveform 4 (Figure 5(b8)) were performed one by one based on the occurrence and magnitude, respectively, of the maximum correlation coefficients (Figure 5(a4–a8)). After this step, the maximum correlation coefficient occurred in channel 3 once again (Figure 5(a9)). Hence, 0.8× and 0.4× subtractions of waveform 3 were tried (Figure 5(b9,b10)); however, both generated a large negative correlation coefficient between the remaining signal and the template waveforms, indicating an over-subtraction had occurred (Figure 5(a10,a11)). As a result, a 0.2× waveform 3 was subtracted (Figure 5(b11)). The remaining signal was then correlated with all template waveforms, and the maximum correlation coefficient (*max*(*r_xy_*) > 0.4) was observed in channel 2 (Figure 5(a12)). Similar to before, 0.8× and 0.4× subtractions were tried (Figure 5(b12,b13)); highly negative maximum correlation coefficients were observed (Figure 5(a13,a14)). Hence, an 0.2× waveform 2 was subtracted (Figure 5(b14)). After this, the maximum correlation coefficient became positive and still occurred in channel 2 (Figure 5(a15)). Therefore, a 0.1× waveform 2 was subtracted (Figure 5(b15)). After the subtraction, the maximum correlation coefficient (>0.4) still occurred in channel 2 (Figure 5(a16)). An 0.05× waveform 2 was subtracted (Figure 5(b16)). Afterwards, the maximum correlation coefficient was positive but below 0.4 (Figure 5(a17)); the interactive cancellation was completed. From the overall subtracted magnitudes of channel 2 (1.15×), channel 3 (2.6×), and channel 4 (2.4×), the particles’ sizes were estimated to be 15.7 μm, 20.6 μm, and 20.1 μm, respectively. The entire interactive cancellation procedure was automatic with a MatLab code and took about 1 ms. It is important to note that although an initial 0.8× subtraction was employed for the magnitude of the iterative cancellation process, aiming to minimize the interaction steps and attain a decent resolution for particle sizing, we also conducted the interactive cancellations with both 0.5× and 0.3× initial subtractions. The obtained particle counts and sizes were nearly identical to those obtained with the 0.8× subtraction.

### 3.2. Demonstration of Sizing and Counting Accuracy

To evaluate the sizing and counting accuracy of the microfluidic sensor, mixtures of particles with different sizes were used. Specifically, solutions containing two differently sized polystyrene microparticles (15 μm and 20 μm) at varying concentrations were prepared by dilution and loaded to the device. While the particle solution flowed through the device, the output signals were recorded across the pair of common electrodes. The output signal was demodulated using the interactive cancellation procedure shown in Figure 4.

**Figure 6:**
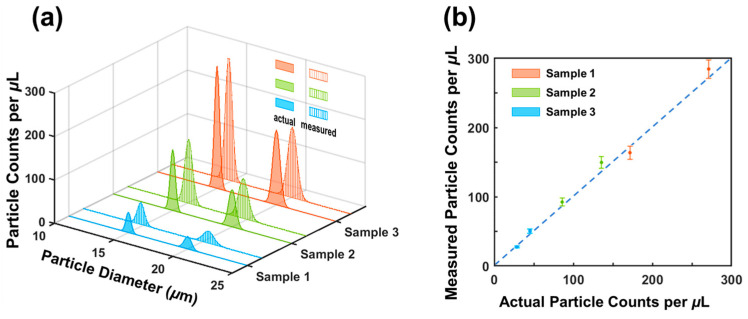
(**a**) Comparison of measured and actual concentrations and diameters of microparticles in the mixed solutions. (**b**) Comparison of measured particle concentrations vs. actual particle concentrations. RPS measurements are represented by small solid circles with error bars. Actual particle concentrations and sizes were measured by an AccuSizer^TM^ 780 (optical particle sizer).

The resulting particle concentrations and sizes of measurement are depicted in Figure 6 (indicated by vertical lines). For sample 1, the measured concentrations were 27.5/mL ± 1.5/mL (for 20 μm) and 50.5/mL ± 3.1/mL (for 15 μm particles); for sample 2, the measured concentrations were 92.4/mL ± 5.3/mL (for 20 μm particles) and 149.6/mL ± 8.5/mL (for 15 μm particles); for sample 3, the measured concentrations were 163.7/mL ± 9.6/mL (for 20 μm particles) and 284.6/mL ± 13.2/mL (for 15 μm particles). For comparison, actual concentrations of the three samples were measured using an AccuSizer^TM^ 780 (optical particle sizer) and are shown in Figure 6a. Encouragingly, the measured concentrations aligned well with the actual concentrations. Figure 6b shows a quantitative comparison between the measurement results and the estimated concentrations. The maximum difference is 5.9% (for 20 μm particles) from sample 3 and 6.1% (for 15 μm particles) from sample 1. The two sets of concentrations matched reasonably well with each other. The small errors in counting may have been caused by the settlement of a small number of cells, considering the polystyrene particles have a slightly higher density than the medium solution.

The sizes of particles were also obtained from the interactive cancellation. In sample 1, the measured particle sizes were 20.5 μm ± 0.4 μm (for 20 μm particles) and 14.8 μm ± 0.5 μm (for 15 μm polystyrene particles); in sample 2, the measured particle sizes were 19.7 μm ± 0.6 μm and 15.1 μm ± 0.4 μm, respectively; in sample 3, the measured sizes were 20.1 μm ± 0.7 μm and 14.7 μm ± 0.5 μm, respectively. The average of the three sizes of measurements was 20.1± 0.6 μm. We also measured the particle sizes using a particle analyzer (AccuSizer^TM^ 780). The particle sizes were measured to be 14.95 μm ± 0.57 μm and 19.6 μm ± 0.74 μm. The RPS measurement and the AccuSizer measurement were in good agreement. The difference was 2.6%. Electrical noise originating from flow fluctuations may have caused errors in the size measurements, as the particle sizes were correlated to the magnitude of the output signal. 

The above results demonstrated the capability of bipolar pulse-width multiplexed RPS to accurately measure the sizes and counts of microparticles in a mixed solution. The sizing of particles was accomplished through the interactive cancellation process, which relied on correlation analysis (maximum correlation coefficient). The correlation analysis indicated the time the maximum correlation coefficient occurred. This information makes it possible to determine the start and ending points of each pulse [28], even if several particles are present in different sensing channels at the same time. 

**Figure 7:**
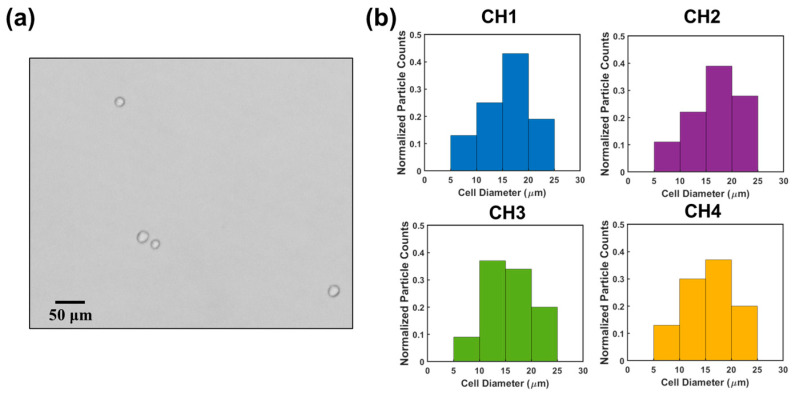
(**a**) HeLa cell image under a microscope. (**b**) Histograms of cell diameter distribution measured across channel 1, channel 2, channel 3, and channel 4.

Finally, we used the sensor to count HeLa cells. HeLa cells are human negroid cervix epithelioid carcinoma cells. These cells play a crucial role in studying the propagation status of cells and advancing our understanding of cancer and viral infections [29,30]. HeLa cells were cultured in DPBS solution (see details in Section 2.1). One HeLa cell sample was loaded to the RPS sensor. The concentration and the size of the HeLa cells were measured to be 0.98 × 10^5^ mL^−1^ and 15.9 ± 3.9 µm (sizes ranging from 8.5 µm to 25.4 µm) using an AccuSizer^TM^ 780. Output signals were collected, which were subsequently demultiplexed by interactive cancellation. Figure 7b shows the cell analysis results in the four sensing channels. The measured concentration of the HeLa cells was approximately (0.93 ± 0.12) × 10^5^ mL^−1^ (obtained from three measurements), which matched with the actual concentration reasonably well. The difference is likely because a small portion of cells settled on the substrate of the channels or attached to the channel walls. This method achieved an accuracy of 94.9% for HeLa cell counting. Compared to the counting accuracies of other electrical multiplexing methods, including the frequency division multiplexing method (i.e., 88%) [22] and CODES method (i.e., 96.15%) [20], this method exhibited a higher or comparable counting accuracy. In terms of particle sizing, this method had a 97.4% accuracy, which is higher than that of the frequency division multiplexing method (i.e., 94.8%). The sizes of the HeLa cells were measured to be 16.6 ± 4.6 µm (ranging from 8.9 μm to 24.5 μm), which were in good agreement with the measured values from the AccuSizer^TM^ 780.

The bipolar pulse-width multiplexing method enables high-throughput microparticle counting and sizing without the need for complex electrode designs or intricate geometry configurations of sensing channels. Only one DC power source, one pair of common electrodes, and one central electrode are required to encode the detected signal. This not only reduces the complexity of the detection electronics but also decreases the data size. While a four-channel device is presented for demonstration of high-throughput particle analysis, the throughput can be further improved by incorporation of additional sensing channels. This method can be extended to nanoscale particle counting, including proteins, nucleic acids, and viruses, by fabricating openings at different positions to expose the central electrode inside the sensing channels. Compared to other multiplexing methods, this fabrication process is simple. It can be extended to measure nanoscale particles by fabricating smaller sensing channels with openings at different locations. Note that the silver coil in the multiplexed RPS can be replaced by sputtering a thin silver film. In addition, this multiplexed RPS can be combined with many well-established antibody- or aptamer-based bio-recognition methods to detect various of micro and nano bio-objects including cells [31,32] and biomolecules [33,34,35] with high throughput and high specificity. 

## 4. Conclusions

We developed a bipolar pulse-width multiplexing-based resistive pulse sensor capable of high-throughput counting and sizing of microparticles through the utilization of multiple parallel sensing channels. The unique bipolar pulse-width multiplexing facilitated the encoding of electrical signals generated by the passage of microparticles, while only requiring one DC source. The combined signal can be demodulated using correlation analysis and an iterative cancellation scheme. This multiplexed RPS was demonstrated using mixtures of differently sized microparticles with varying concentrations. The RPS sensor can predict the sizes and concentrations of standard polystyrene particles accurately with errors of 2.6% and 6.1% in sizing and counting, respectively. The sensor was demonstrated to accurately count HeLa cells, with an error of 5.6% in concentration. Due to the simple fabrication process, smaller sensing channels can be fabricated for detection and analysis of nanoscale analytes. With its high throughput and accuracy, this RPS sensor holds promise for the rapid analysis of micro and nano objects including biomolecules, viruses, and bacteria, especially in resource-limited environments.

## Data Availability

Data will be made available on request.

## References

[1] Holzinger M., Le Goff A., Cosnier S. (2014). Nanomaterials for biosensing applications: A review. Front. Chem..

[2] Tansil N.C., Gao Z. (2006). Nanoparticles in biomolecular detection. Nano Today.

[3] Ventura-Aguilar R.I., Bautista-Baños S., Mendoza-Acevedo S., Bosquez-Molina E. (2023). Nanomaterials for designing biosensors to detect fungi and bacteria related to food safety of agricultural products. Postharvest Biol. Technol..

[4] Pividori M.I., Alegret S. (2010). Micro and nanoparticles in biosensing systems for food safety and environmental monitoring. An example of converging technologies. Microchim. Acta.

[5] Xianyu Y., Wang Q., Chen Y. (2018). Magnetic particles-enabled biosensors for point-of-care testing. TrAC Trends Anal. Chem..

[6] Kozak D., Anderson W., Vogel R., Trau M. (2011). Advances in resistive pulse sensors: Devices bridging the void between molecular and microscopic detection. Nano Today.

[7] Weatherall E., Willmott G.R. (2015). Applications of tunable resistive pulse sensing. Analyst.

[8] DeBlois R.W., Bean C.P. (1970). Counting and Sizing of Submicron Particles by the Resistive Pulse Technique. Rev. Sci. Instrum..

[9] Song Y., Zhang J., Li D. (2017). Microfluidic and Nanofluidic Resistive Pulse Sensing: A Review. Micromachines.

[10] Yang L., Yamamoto T. (2016). Quantification of Virus Particles Using Nanopore-Based Resistive-Pulse Sensing Techniques. Front. Microbiol..

[11] Vaclavek T., Prikryl J., Foret F. (2019). Resistive pulse sensing as particle counting and sizing method in microfluidic systems: Designs and applications review. J. Sep. Sci..

[12] Zhou T., Song Y., Yuan Y., Li D. (2019). A novel microfluidic resistive pulse sensor with multiple voltage input channels and a side sensing gate for particle and cell detection. Anal. Chim. Acta.

[13] Pan R., Hu K., Jia R., Rotenberg S.A., Jiang D., Mirkin M.V. (2020). Resistive-Pulse Sensing Inside Single Living Cells. J. Am. Chem. Soc..

[14] Luo L., German S.R., Lan W.-J., Holden D.A., Mega T.L., White H.S. (2014). Resistive-Pulse Analysis of Nanoparticles. Annu. Rev. Anal. Chem..

[15] Sikora A., Shard A.G., Minelli C. (2016). Size and Zeta-Potential Measurement of Silica Nanoparticles in Serum Using Tunable Resistive Pulse Sensing. Langmuir.

[16] Blundell E.L.C.J., Mayne L.J., Billinge E.R., Platt M. (2015). Emergence of tunable resistive pulse sensing as a biosensor. Anal. Methods.

[17] Sivakumaran M., Platt M. (2016). Tunable resistive pulse sensing: Potential applications in nanomedicine. Nanomedicine.

[18] Song Y., Yang J., Pan X., Li D. (2015). High-throughput and sensitive particle counting by a novel microfluidic differential resistive pulse sensor with multidetecting channels and a common reference channel. Electrophoresis.

[19] Liu R., Waheed W., Wang N., Civelekoglu O., Boya M., Chu C.-H., Sarioglu A.F. (2017). Design and modeling of electrode networks for code-division multiplexed resistive pulse sensing in microfluidic devices. Lab Chip.

[20] Liu R., Wang N., Kamili F., Sarioglu A.F. (2016). Microfluidic CODES: A scalable multiplexed electronic sensor for orthogonal detection of particles in microfluidic channels. Lab Chip.

[21] Xu R., Ouyang L., Shaik R., Zhang G., Zhe J. (2023). Multiplexed resistive pulse sensor based on geometry modulation for high-throughput microparticle counting. Sens. Actuators Rep..

[22] Jagtiani A.V., Carletta J., Zhe J. (2011). A microfluidic multichannel resistive pulse sensor using frequency division multiplexing for high throughput counting of micro particles. J. Micromech. Microeng..

[23] Dinan E., Jabbari B. (1998). Spreading codes for direct sequence CDMA and wideband CDMA cellular networks. IEEE Commun. Mag..

[24] Schober P., Boer C., Schwarte L.A. (2018). Correlation Coefficients: Appropriate Use and Interpretation. Anesth. Analg..

[25] Akoglu H. (2018). User’s guide to correlation coefficients. Turk. J. Emerg. Med..

[26] Mukaka M.M. (2012). Statistics corner: A guide to appropriate use of correlation coefficient in medical research. Malawi Med. J..

[27] Wu X., Chon C.H., Wang Y.-N., Kang Y., Li D. (2008). Simultaneous particle counting and detecting on a chip. Lab A Chip.

[28] Shen C. (2015). Analysis of detrended time-lagged cross-correlation between two nonstationary time series. Phys. Lett. A.

[29] Strauss N., Hendee E.D. (1959). The effect of diphtheria toxin on the metabolism of Hela cells. J. Exp. Med..

[30] Girardi A.J., Mc Michael H., Henle W. (1956). The use of HeLa cells in suspension for the quantitative study of virus propagation. Virology.

[31] Saleem A., Husheem M., Härkönen P., Pihlaja K. (2002). Inhibition of cancer cell growth by crude extract and the phenolics of *Terminalia chebula* retz. fruit. J. Ethnopharmacol..

[32] Xu R., Ouyang L., Chen H., Zhang G., Zhe J. (2023). Recent Advances in Biomolecular Detection Based on Aptamers and Nanoparticles. Biosensors.

[33] Xu R., Abune L., Davis B., Ouyang L., Zhang G., Wang Y., Zhe J. (2022). Ultrasensitive detection of small biomolecules using aptamer-based molecular recognition and nanoparticle counting. Biosens. Bioelectron..

[34] Billinge E.R., Broom M., Platt M. (2014). Monitoring Aptamer–Protein Interactions Using Tunable Resistive Pulse Sensing. Anal. Chem..

[35] Billinge E.R., Platt M. (2015). Multiplexed, label-free detection of biomarkers using aptamers and Tunable Resistive Pulse Sensing (AptaTRPS). Biosens. Bioelectron..

